# Enhanced osteogenic potential of mesenchymal stem cells from cortical bone: a comparative analysis

**DOI:** 10.1186/s13287-015-0193-z

**Published:** 2015-10-26

**Authors:** Joseph S. Fernandez-Moure, Bruna Corradetti, Paige Chan, Jeffrey L. Van Eps, Trevor Janecek, Pranela Rameshwar, Bradley K. Weiner, Ennio Tasciotti

**Affiliations:** Houston Methodist Hospital Department of Surgery, Houston, USA; Department of Nanomedicine, Houston Methodist Research Institute, 6670 Bertner Avenue, Houston, TX 77030 USA; Department of Life and Environmental Sciences, Università Politecnica delle Marche, via Brecce Bianche, 60131 Ancona, Italy; Houston Methodist Hospital Department of Orthopedic Surgery, 6565 Fannin Street, Houston, TX 77030 USA; Department of Medicine, Rutgers New Jersey Medical School, 185 South Orange Avenue, Newark, NJ 07103 USA

**Keywords:** Cortical bone, Mesenchymal stem cells, Multipotent differentiation ability, Osteogenic potential, Hypoxia, Normoxia

## Abstract

**Introduction:**

Mesenchymal stem cells (MSCs) hold great promise for regenerative therapies in the musculoskeletal system. Although MSCs from bone marrow (BM-MSCs) and adipose tissue (AD-MSCs) have been extensively characterized, there is still debate as to the ideal source of MSCs for tissue-engineering applications in bone repair.

**Methods:**

MSCs were isolated from cortical bone fragments (CBF-MSCs) obtained from patients undergoing laminectomy, selected by fluorescence-activated cell sorting analysis, and tested for their potential to undergo mesodermic differentiation. CBF-MSCs were then compared with BM-MSCs and AD-MSCs for their colony-forming unit capability and osteogenic potential in both normoxia and hypoxia. After 2 and 4 weeks in inducing media, differentiation was assessed qualitatively and quantitatively by the evaluation of alkaline phosphatase (ALP) expression and mineral deposition (Von Kossa staining). Transcriptional activity of osteoblastogenesis-associated genes (*Alp*, *RUNX2*, *Spp1*, and *Bglap*) was also analyzed.

**Results:**

The cortical fraction of the bone contains a subset of cells positive for MSC-associated markers and capable of tri-lineage differentiation. The hypoxic conditions were generally more effective in inducing osteogenesis for the three cell lines. However, at 2 and 4 weeks, greater calcium deposition and ALP expression were observed in both hypoxic and normoxic conditions in CBF-MSCs compared with AD- and BM-MSCs. These functional observations were further corroborated by gene expression analysis, which showed a significant upregulation of *Bglap*, *Alp*, and *Spp1*, with a 22.50 (±4.55)-, 46.56 (±7.4)-, 71.46 (±4.16)-fold increase compared with their uninduced counterparts.

**Conclusions:**

This novel population of MSCs retains a greater biosynthetic activity *in vitro*, which was found increased in hypoxic conditions. The present study demonstrates that quantitative differences between MSCs retrieved from bone marrow, adipose, and the cortical portion of the bone with respect to their osteogenic potential exist and suggests the cortical bone as suitable candidate to use for orthopedic tissue engineering and regenerative medicine.

## Introduction

Mesenchymal stem cells (also known as multipotent stromal cells, or MSCs) are a group of cells defined for their capacity to self-renew and differentiate toward the mesodermal lineage, becoming osteoblasts, adipocytes, and chondrocytes [1–3]. MSCs from the bone marrow (BM-MSCs) were originally described by Friedenstein et al. as non-phagocytic and non-hematopoietic cells and adherent to plastic [4]. Over the last 15 years, there has been an explosion in the reports of MSCs isolated from a variety of other adult sources, including skin [5], adipose tissue [6], umbilical cord blood [7, 8] and matrix, peripheral blood [9], tendons [10, 11], amnion [12–14], and bone [15–17]. Their plasticity, immunosuppressive potential, immuno-modulatory properties [18, 19], and trophic activity [20, 21] make MSCs critical players in tissue homeostasis. For these reasons, MSCs are considered a suitable tool for regenerative medicine and have been already introduced in a number of clinical trials for tissue repair [18, 22–25]. Although they share similar epitope profiles, MSCs derived from different tissues show significant differences in the differentiation, proliferation, and migration potential, which depend on the tissue they originate from as they receive inputs that directly affect their specification [2, 26, 27].

For regenerative medicine purposes, BM-MSCs represent the gold standard [4, 22–25] and their use in investigational and clinical orthopedic tissue engineering has been well characterized [18–20]. Following much debate, however, MSCs with true “stemness” have been shown to constitute only a very small proportion of cells in the bone marrow (0.01–0.001 % of nucleated cells) and their proliferative and differentiative potential inversely correlate with age and the passages *in vitro* [21, 28]. Furthermore, bone marrow aspiration is painful and can be associated with multiple complications [29]. Adipose tissue has arisen as a reliable source for MSCs [30, 31]. They can be obtained by the less invasive method of lipoaspiration and yield a greater quantity of tissue and thus a greater number of cells [26, 27, 32, 33]. AD-MSCs have been shown to have a greater potential for proliferation, higher rates of colony formation, and greatest tolerance to serum deprivation-induced apoptosis than their bone marrow counterparts [28, 34–37].

Although BM-MSCs and AD-MSCs have been extensively characterized, there is still debate regarding the ideal source of MSCs for orthopedic tissue-engineering applications [18, 35]. Orthopedic reconstructive procedures remain some of the most common procedures performed worldwide. Historically, surgical reconstruction has been predominated by the use of synthetic implants and bone grafts. Over the past 15 years, there has been an explosion in the use of cell-based therapies in orthopedics [38–40]. Although BM-MSCs are the most commonly used for this purpose, groups have described different compartments of the bone, mainly the trabecular [16, 41–43] and cortical [17, 44] portions, as reservoirs of multipotent cells with a greater osteogenic commitment [15, 44, 45]. Moreover, their characterization has been limited to animal models and no comparison in humans has yet been reported. The aims of the present study were to isolate and characterize native populations of MSCs from the human cortical bone fraction (CBF-MSCs) and to compare them with commercially available MSCs obtained from the two most studied sources: the adipose tissue and the bone marrow. Comparison was performed in terms of morphology, clonogenic capability (colony-forming units, or CFU), and multidifferentiative potential, with particular emphasis to the osteogenic commitment, which has been assessed through the evaluation of calcium deposition, alkaline phosphatase (ALP), and osteoblastogenesis-associated genes expression.

## Methods

### Isolation of human CBF-MSCs

CBF-MSC populations were extracted by processing bones of three patients undergoing laminectomy following a protocol previously described [17] with some modifications for humans. All three patients were age-matched and all underwent the same procedure, spinal laminectomy, for benign pathology. This extraction protocol was approved by the Institutional Review Board of the Houston Methodist Hospital (Houston, TX, USA). Samples were processed after written informed consent was obtained. Bone fragments were obtained following the routine dissection and extraction of bone as part of the standard operation. No modification to the original surgery was made for the removal of bone fragments. The fragments used would have otherwise been discarded and no excess tissue that would have not been removed as standard of care for the planned procedure was extracted.

Bones were cleaned of any fat, connective tissue, or muscle by sharp dissection. Any blood that remained on the specimen was flushed from the bony fragments with phosphate-buffered saline (PBS) (Thermo Fisher Scientific, Waltham, MA, USA) supplemented with 1 % antibiotic/antimycotic (Gibco, Grand Island, NY, USA) until they appeared clean of all debris. The cancellous portion of the bone was sharply transected from the cortical fraction and discarded. Cortical bone fragments were crushed into chips approximately 3–4 mm^3^ and transferred to 50-ml polypropylene tubes (BD Falcon, Bedford, MA, USA). Bone chips were suspended in alpha-modified Eagle’s medium (α-MEM) containing 2 % (vol/vol) defined fetal bovine serum (FBS) (Gibco) in presence of 3 mg/ml collagenase type-I (Worthington Biochemical Corporation, Lakewood, NJ, USA) and 4 mg/ml dispase II (Roche, Indianapolis, IN, USA) and placed on a shaking platform at 37 °C for 3 h. Following digestion, bone chips were plated into new flasks and grown undisturbed for 3 days to allow cells to migrate out of the fragments. A mixture of cells obtained from digestion and migration from the bone chips was obtained. These cells were considered passage 0 (P0).

### Histological staining of bone fragments

To assess the complete removal of cells from the bone matrix, histologic staining was performed. Bone fragments underwent enzymatic processing as mentioned above. Before enzymatic digestion and after migration of cells from the bone fragments, representative fragments were fixed in 10 % buffered formalin and then underwent decalcification in an 8 % hydrochloric acid/formic acid solution. Tissue samples were then embedded in paraffin and cut into 4-μm sections using a microtome. Three non-consecutive sections were routinely stained with hematoxylin and eosin (H&E) and inspected for presence of cells following digestion.

### CBF-MSC expansion

Adherent cells were cultured in α-MEM (Sigma-Aldrich, St. Louis, MO, USA) containing 20 % (vol/vol) FBS supplemented with 1 % antibiotic/antimycotic (Gibco) and incubated at 37 °C in hypoxic (5 % oxygen) or normoxic (21 % oxygen) conditions and 90 % humidity. Media was changed every 48 h until the cells were at 60–80 % confluency at which point they were passaged at a split ratio of 1:3. Cultures were established in Dulbecco’s modified Eagle’s medium (DMEM) containing 10 % (vol/vol) FBS supplemented with 1 % antibiotic/antimycotic (Gibco). AD- and BM-MSC cultures were established in accordance with the instructions of the manufacturer (Lonza, Basel, Switzerland). Adherent P2 cells were serially passaged by using TripLE™ Express (Invitrogen, part of Thermo Fisher Scientific) upon reaching near confluence (80 %) and reseeded for culture maintenance.

To confirm the MSC-associated phenotype of cells obtained from the cortical portion of the bone and work with a homogeneous cell population, freshly isolated CBF-MSCs were selected by fluorescence-activated cell sorting (FACS) based on a panel of lineage-committed cell surface markers.

### Fluorescence-activated cell sorting

Approximately 1 × 10^6^ cells were collected and stained in accordance with guidelines of the manufacturer. Cells used in FACS were at P0. Tested markers included the 5′-nucleotidase, CD73 (BioLegend, San Diego, CA, USA), the receptor-linked protein tyrosine phosphatase, CD45 (BioLegend), B-lymphocyte antigen CD19 (eBioscience, San Diego, CA, USA), the major histocompatibility complex (MHC) class II cell surface receptor, HLA-DR (BioLegend), the surface glycoprotein and cell-cell adhesion factor, CD34 (Invitrogen, Burlington, ON, Canada), the glycoprotein CD44 (Invitrogen), and membrane glycoprotein, CD105 (Invitrogen). Cells (5 × 10^5^) were incubated with directly conjugated antibodies in 0.1 % bovine serum albumin/PBS for 30 min at room temperature in the dark. Cells were first gated on the basis of light-scatter properties to screen out debris. MSC cell surface phenotypes were verified through a multiparameter panel permitting the selection of CD73^+^CD44^+^CD105^+^HLA-DR^−^CD19^−^CD34^−^ cells on a BD FACSAria™ II cell sorter (Becton Dickinson, Franklin Lakes, NJ, USA) to obtain a near-pure subpopulation of CBF-MSCs. Sorted cells were collected and cultured to perform proliferation assays, colony-forming unit-fibroblastic-like (CFU-F) analysis, and multilineage differentiation.

### Proliferation assays (doubling time) in normoxic and hypoxic conditions

Proliferation rate was determined in triplicate on AD-, BM-, and CBF-MSCs as previously reported [46, 47]. Doubling time was assessed from P1 to P10 in normoxic and hypoxic conditions to determine the effect of low-tension oxygen on cell proliferation. Data obtained from each cell line are reported as mean of the values. For CBF-MSCs, the doubling time at each passage has also been shown.

#### Colony-forming unit (CFU-F) assay

Colony-forming unit assay was performed on freshly isolated CBF-MSCs, AD-MSCs, and BM-MSCs as previously reported [12, 17, 48]. Briefly, cells at P2 were plated in six-well plates at different densities (100, 250, 500, and 1000 cells/cm^2^) and cultured over a 15-day period. Colonies were fixed with 4 % formalin, stained with 1 % methylene blue (Serva, Heidelberg, Germany) in 10 mM borate buffer pH 8.8 (Fluka BioChemika, Buchs, Switzerland) at room temperature, and washed twice. Colonies formed by 16–20 nucleated cells were counted under a BX71 microscope (Nikon Corporation, Tokyo, Japan).

### Adipogenic, chondrogenic, and osteogenic differentiation

Multipotent differentiation capability of CBF-MSCs, AD-MSCs, and BM-MSCs was assessed *in vitro* at P3. AD-MSCs and BM-MSCs were purchased from (Lonza) and delivered at P0. They were expanded to P3 in DMEM containing 10 % (vol/vol) FBS and used for subsequent differentiation assays. Non-induced AD-MSCs, BM-MSCs, and CFB-MSCs were used as control and cultured for the same time in growth medium.

To assess chondrogenic differentiation, micromass cultures were generated by seeding 5-μl droplets of a 1.6 × 10^7^cells/ml solution into the center of a multi-well culture vessel. The micromass was allowed to settle for 2 h and then complete StemPro replaced with complete StemPro Media (Gibco) was gently added so as to not perturb the micromass. Cells were allowed to undergo differentiation for 21 days with media change every 2 days. After induction, micromass cultures were stained by Alcian blue stain for glycosaminoglycans and mucopolysaccharides.

Cells undergoing adipogenic differentiation were seeded into culture vessels at the density of 1 × 10^4^ cells/cm^2^ and incubated for 24 h in standard medium. Then the medium was replaced with complete StemPro Adipogenic Differentiation medium (Gibco) and incubated for 14 days. Media were changed every 3 days. After induction, cells were stained by Oil red O to highlight the presence of intracellular lipid vacuoles. Osteogenic induction was performed by seeding cells at the density of 5000 cells/cm^2^ in 12-well plates and culturing them until they reached approximately 80–90 % confluence.

The osteogenic potential of cells cultured in hypoxic or normoxic conditions was assessed *in vitro*. In both cases, induction was performed over 2 and 4 weeks by using StemPro Osteogenesis Differentiation Madium (Gibco). To confirm mineral deposition, conventional von Kossa and ALP stainings were performed by using a Vector Blue Alkaline Phosphatase Substrate Kit (Vector Labs, Burlingame, CA, USA). Equal numbers of cells were plated in eight-well plates and grown for 2 weeks in both hypoxia and normoxic conditions. Staining was then performed in accordance with the instructions of the manufacturer. Fluorescence intensity per field of view was quantified by using ImageJ software, and total fluorescence was normalized to cell number. Five fields of view per well were quantified. Similarly, cells were grown in hypoxic and normoxic condition for 2 and 4 weeks and then stained by Von Kossa. Five fields of view were chosen for analysis, and the area of positively staining material was quantified by using ImageJ software.

### Molecular characterization

Quantitative reverse transcription-polymerase chain reaction analysis was used to evaluate the expression of the precursor-associated gene *CD271* and specific osteogenesis-associated markers following induction. Total RNA was isolated from CBF-MSCs, AD-MSCs, and BM-MSCs by using Trizol reagent (Invitrogen). DNAse (Sigma-Aldrich) treatment followed the reaction. RNA concentration and purity were measured by using a NanoDrop ND1000 spectrophotometer (NanoDrop Technologies, Wilmington, DE, USA). The cDNA was synthesized from 1 μg total RNA by using an iScript retrotranscription kit (Bio-Rad Laboratories, Hercules, CA, USA), and quantitative polymerase chain reaction was run in an ABI 7500 Fast Sequence Detection System (Applied Biosystems, Foster City, CA, USA) using commercially available master mix. The following target probes (Applied Biosystems) were used to evaluate the expression of Runt-related transcription factor 2 (*RUNX2*: Hs00231692_m1) osteocalcin (*Bglap*; Hs01587814_g1), osteopontin (*Spp1*; Hs00959010_m1), and ALP (*Alp*; Hs01029144_m1) expression for osteogenesis. The expression of each gene was normalized to the level of glyceraldehyde 3-phosphate dehydrogenase (*Gapdh*; Hs02758991_g1) and presented in comparison with the values obtained from the control (uninduced cells). For the expression of CD271 (Hs00609977_m1), CBF-MSCs were compared with MSCs obtained from their bone-marrow counterparts.

### Statistical analysis

All experiments were repeated a minimum of three times, and all data are presented as mean ± standard deviation. All statistical calculations were completed using GraphPad Prism software (GraphPad Software, Inc., La Jolla, CA, USA). A paired *t* test was used to analyze the statistical significance of hypoxic against normoxic conditions per cell line and to determine the statistical significance of one hypoxic cell line to another. Anything with a *P* value of less than 0.05 was considered statistically significant.

## Results

### CBF-MSC collection and morphology

**Fig. 1:**
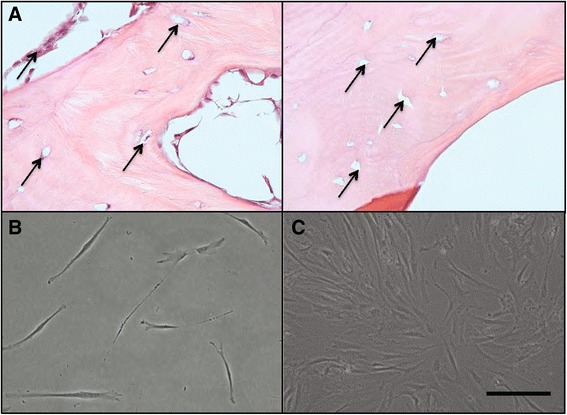
**a** Hematoxylin-and-eosin images showing the cortical portion of the bone before (*left side*) and after (*right side*) digestion. *Black arrows* indicate canaliculi. Fibroblast-like cells at 72 h (**b**) and at 5 days (**c**) after the initial culture. Magnification: 20×. Scale bar: 40 μm

As shown by H&E stainings, after digestion with dispase and collagenase I, the bone fragments had little to no cells or cellular debris visible (Fig. 1a). Spindle-shaped cells appeared following isolation (Fig. 1b), and a confluent layer of cells was seen within 21 days following primary cell culture (Fig. 1c).

### Fluorescence-activated cell sorting analysis

**Fig. 2:**
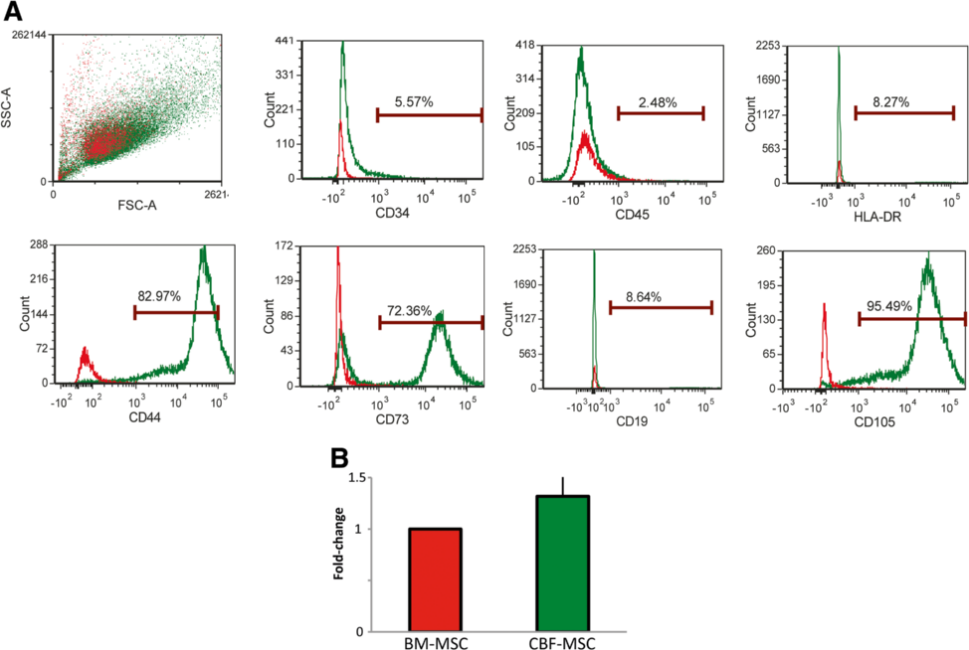
Immunophenotyping characterization. **(a)** Flow cytometric analysis showing morphological plots for CBF-MSCs soon after isolation (passage 0). Cells obtained from the cortical portion of the bone represent a homogeneous population that stains positively for mesenchymal stem cell-associated markers (CD44, CD73, and CD105) and negative for hematopoietic (CD45 and CD34)- and the major histocompatibility complex-class II (HLA-DR)- or B-lymphocyte antigen (CD19)-associated markers. Stained cells are represented in *green*, whereas unstained cells seen in *red* were used as controls. (**b**) Quantitative polymerase chain reaction analysis of RNA expression of the common progenitor-associated marker CD271 in BM- and CBF-MSCs. Data are represented as fold-change compared with BM-MSCs. Values are mean ± standard deviation (*n* = 3). *BM-MSC* bone marrow mesenchymal stem cell, *CBF-MSC* cortical bone fragment mesenchymal stem cells

In Fig. 2, the plot of our protocol allowed for the isolation of a heterogeneous cell population with a preponderance of cells displaying phenotypically similar surface markers from the cortical portion of the bone. Within this population, FACS analysis demonstrated that more than 72, 82, and 95 % of cells derived from CBF-MSCs stained positively for the typical MSC marker antigens CD73, CD44, and CD105, respectively (Fig. 2a). Sorting was done by combination staining and multicolor analysis to allow us to see whether cells were co-expressing surface markers that were chosen on the basis of previously published reports [14]. The cells from the three different donors were found to be phenotypically similar and thus a representative display of the expression plots is shown in Fig. 2. As for AD-MSCs and BM-MSCs, CBF-MSCs showed no expression of the hematopoietic markers CD34 and CD45, MHC class II maker HLA-DR, or B-lymphocyte antigen CD19. No differences in the expression of the MSC-precursor associated marker CD271 were found in sorted CBF-MSCs in comparison with BM-MSCs (Fig. 2b).

### Proliferation rate in normoxic and hypoxic conditions

**Fig. 3:**
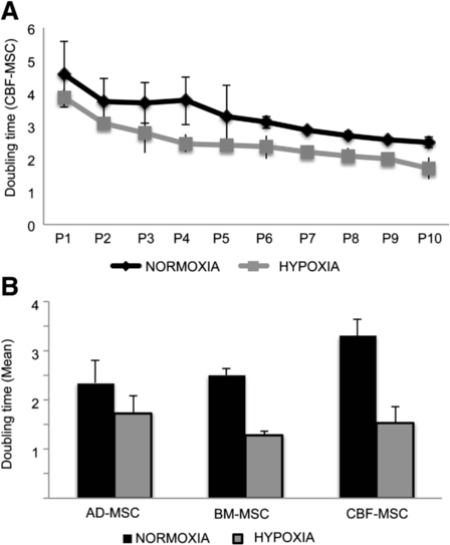
Cell proliferation in normoxic and hypoxic conditions. **a** Graph represents the doubling time in normoxic and hypoxic conditions during cell culture for AD-, BM-, and CBF-MSCs expressed as mean value between data obtained from P1 to P10. **b** Doubling time evaluated on CBF-MSCs at each passage in normoxic and hypoxic conditions. Asterisks represent statistically different doubling time means between normoxia and hypoxia, *n* = 3: *P < 0.05. *AD-MSC* adipose tissue mesenchymal stem cell, *BM-MSC* bone marrow mesenchymal stem cell, *CBF-MSC* cortical bone fragment mesenchymal stem cells, *P* passage

In the doubling time values obtained in CBF-MSCs at each passage (from P1 to P10), the same trend was observed in normoxic and hypoxic conditions, showing a progressive decreasing value. The doubling time for the first passages was higher than the later passages, and mean values dropped from 4.58 ± 1 and 2.5 ± 0.15 days in normoxia and from 3.87 ± 0.25 to 1.69 ± 0.33 days in hypoxia. As expected, the proliferative capacity of CBF-MSCs *in vitro* was generally slower than that observed in BM-MSCs and AD-MSCs in normoxic conditions, where the average values have been addressed around 3.24 ± 0.33, 2.5 ± 0.4, and 2.34 ± 0.45 for CBF-MSCs, BM-MSCs, and AD-MSCs, respectively (Fig. 3b).

#### Colony-forming unit (CFU-F) assay

**Fig. 4:**
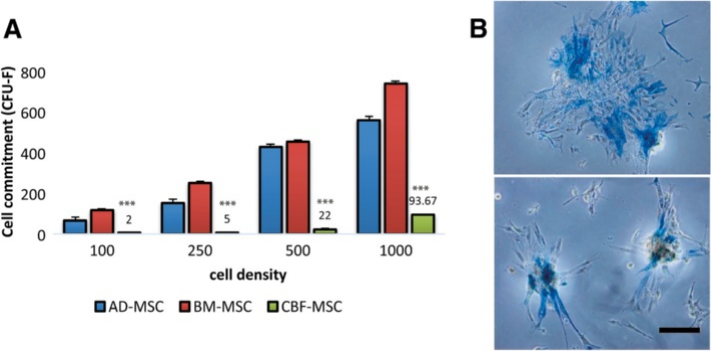
Colony-forming unit (CFU-F) assay. **a** Graph showing the number of CFU-Fs (expressed as a measure of cell commitment) counted in AD-, BM-, and CBF-MSCs when cells were seeded at different densities. A marked reduction (**P < 0.01) in the number of colonies in CBF-MSCs compared with AD- and BM-MSCs. **b** Representative images showing colonies formed by CBF-MSCs that stained positively for alkaline phosphatase. Magnification: 20×. Scale bar: 20 μm. *AD-MSC* adipose tissue mesenchymal stem cell, *BM-MSC* bone marrow mesenchymal stem cell, *CBF-MSC* cortical bone fragment mesenchymal stem cells

When MSC samples were examined and compared for clonogenicity, a significant increase in CFU-Fs was found at higher cell-seeding densities in each of the three cell lines tested (Fig. 4a). However, among AD-, BM-, and CBF-MSCs, statistically significant differences in the clonogenic potential have been found, with CBF-MSCs showing the lowest clonogenicity. CBF-MSCs demonstrated the fewest yet largest CFU-Fs per number of cells plated. In contrast, BM-MSCs and AD-MSCs had three to four times more CFU-Fs detected after plating when applying the same initial plating density. CBF-MSCs were noted to spontaneously differentiate toward the osteogenic lineage and stain positively for ALP (Fig. 4b).

#### Chondrogenic and adipogenic differentiation

**Fig. 5:**
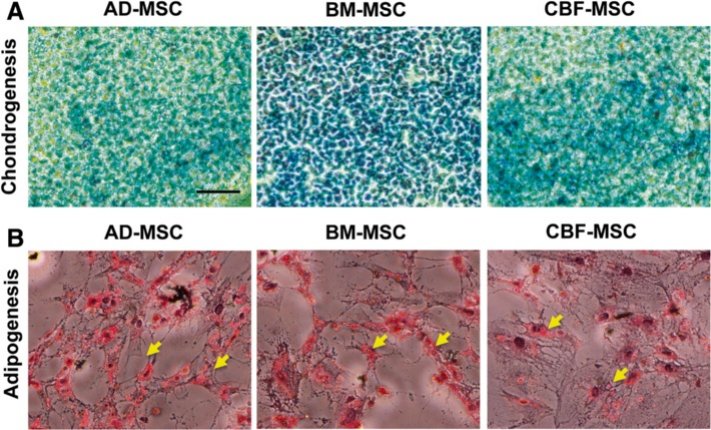
Differentiation assay. **a** Chondrogenic (Alcian blue, 20× magnification) and (**b**) adipogenic (Oil red O, 20× magnification) differentiation of AD-, BM-, and CBF-MSCs. *Yellow arrows* show intracellular lipidic vacuoles that are representative of adipogenic induction. Magnification: 20×. Scale bar: 40 μm. *AD-MSC* adipose tissue mesenchymal stem cell, *BM-MSC* bone marrow mesenchymal stem cell, *CBF-MSC* cortical bone fragment

Chondrogenic differentiation was demonstrated by micromass culture of cells for 21 days in chondrogenic media. After 21 days, all samples, irrespective of their origin, stained positively for Alcian blue, which is known to be a marker for glycosaminoglycans and mucopolysaccharides (Fig. 5a). Adipogenic potential was demonstrated by AD-MSCs, BM-MSCs, or CBF-MSCs in either adipogenic medium over a 14-day period. The three cell populations responded to adipocyte induction by accumulation of positively staining lipid vacuoles in the cytoplasm of the cell (Fig. 5b, *yellow arrows*).

#### Alkaline phosphatase activity

**Fig. 6:**
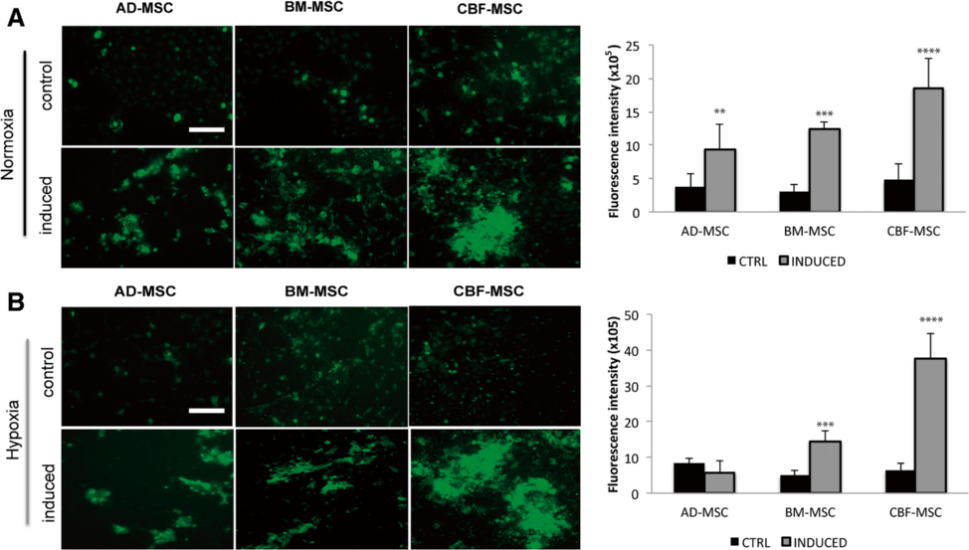
Effects of hypoxia on alkaline phosphatase activity in of AD-, BM-, and CBF-MSCs after 2 weeks in induction media. Fluorescent staining of alkaline phosphatase of induced and non-induced cells (control) of all three types in normoxic (**a**) and hypoxic (**b**) conditions and respective quantification. Values are mean ± standard deviation (*n* = 3). Asterisks represent significant differences between induced AD-, BM, and CBF-MSCs: **P < 0.05, ***P < 0.001, ****P = 0.0004. Magnification: 10×. Scale bar: 40 μm. *AD-MSC* adipose tissue mesenchymal stem cell, *BM-MSC* bone marrow mesenchymal stem cell, *CBF-MSC* cortical bone fragment mesenchymal stem cells, *CTRL* control

To evaluate the osteogenic phenotype of the cell lines, ALP activity was assessed. Because ALP activity is recognized as an early marker of osteoblastic differentiation, it was measured at 2 weeks [49]. ALP activity was greatest in induced CBF-MSCs, and no significant differences were observed between the AD- and BM-MSCs (Fig. 6). Under hypoxic conditions, all cell types demonstrated increased ALP activity (Fig. 6b). Compared with AD- and BM-MSCs, CBF-MSCs showed nearly fourfold greater ALP activity in hypoxic conditions (*P* < 0.05). CBF-MSC ALP activity increased compared with the normoxic counterpart (*P* = 0.0004, Fig. 6a).

#### Von Kossa staining and quantification

**Fig. 7:**
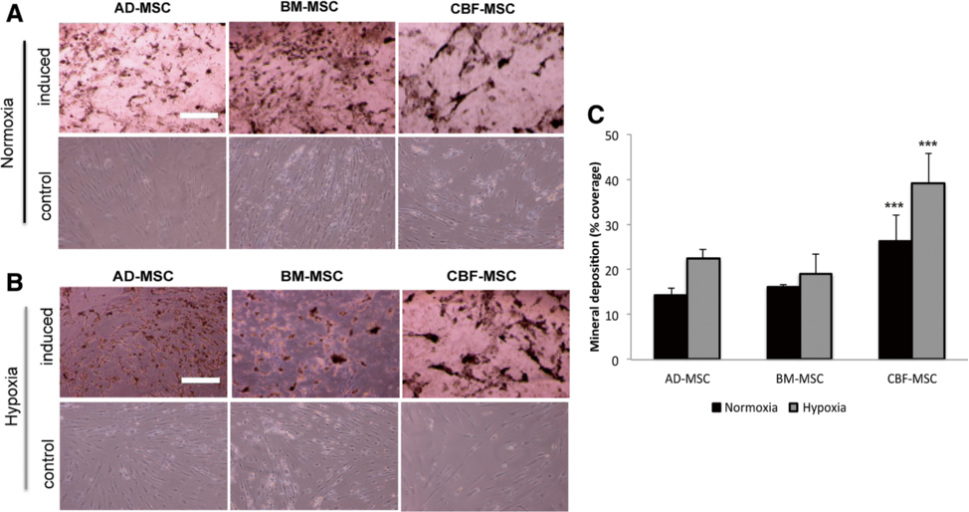
Osteogenic potential assessed on AD-, BM-, and CBF-MSCs after 2 weeks in induction media. Cells were cultured in normoxic (**a**) and hypoxic (**b**) conditions. Histological images showing mineral deposits produced by the three cell types. Cells grown in standard media for the same period are also shown (second and fourth rows). Magnification: 10×. Scale bar: 40 μm. (**c**) Quantification of mineral deposition following osteogenic induction in normoxia and hypoxia conditions represented as a percentage of total well area. Values are mean ± standard deviation (*n* = 3). Asterisks depict highly significant differences (*P* < 0.0005) between CBF-MSCs and AD-MSCs and BM-MSCs. *AD-MSC* adipose tissue mesenchymal stem cell, *BM-MSC* bone marrow mesenchymal stem cell, *CBF-MSC* cortical bone fragment

**Fig. 8:**
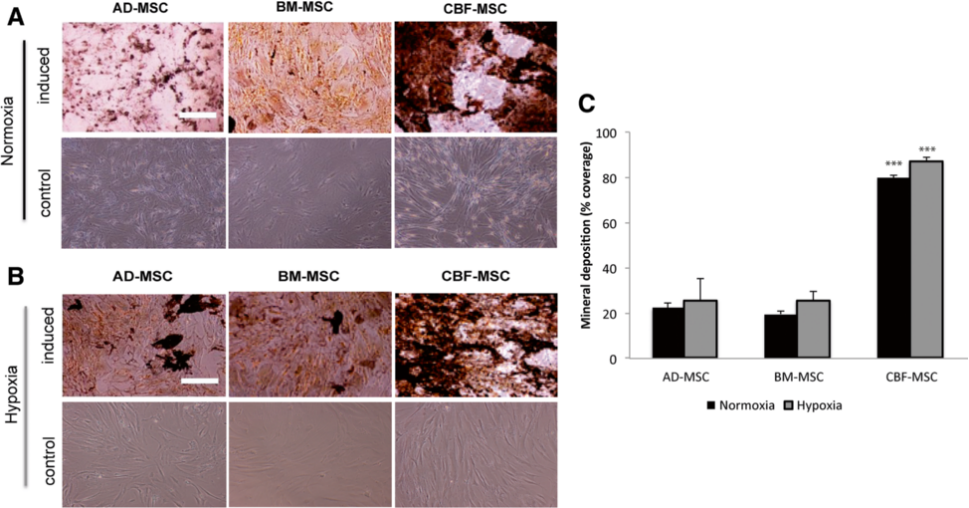
Osteogenic potential assessed on AD-, BM-, and CBF-MSCs after 4 weeks in induction media. Cells were cultured in normoxic (**a**) and hypoxic (**b**) conditions. von Kossa stainings showing mineral deposits produced by the three cell types. Cell grown in standard media for the same period are also showed (second and fourth rows). Magnification: 10×. Scale bar: 40 μm. **c** Quantification of mineral deposition following osteogenic induction in normoxia and hypoxia represented as a percentage of total well area. Values are mean ± standard deviation (*n* = 3). Asterisks depict highly significant differences (*P* < 0.0005) between CBF-MSCs and AD-MSCs and BM-MSCs. *AD-MSC* adipose tissue mesenchymal stem cell, *BM-MSC* bone marrow mesenchymal stem cell, *CBF-MSC* cortical bone fragment mesenchymal stem cells

A greater capability of CBF-MSCs to undergo osteoblastogenesis compared with AD-MSCs and BM-MSCs was demonstrated qualitatively and quantitatively by the formation of mineralized nodules as detected by von Kossa staining in normoxic and hypoxic conditions (Figs. 7 and 8). CBF-MSCs had a greater average amount of calcium deposition at both 2 (Fig. 7a) and 4 (Fig. 8a) weeks. The amount of mineralized nodules seen covering the wells in CBF-MSCs at 4 weeks was significantly greater than the average amount of calcium deposited by AD- and BM-MSCs following exposure to both normoxic and hypoxic conditions (Fig. 8). No significant differences were seen among the AD- and BM-MSCs. CBF-MSCs in either normoxic or hypoxic conditions. CBF-MSCs had greater amounts of mineralization compared with AD- and BM-MSCs (Fig. 8b). The increased calcium deposition exhibited by CBF-MSCs after 4 weeks suggests a continued and greatly enhanced potential for mineralization. Bone marrow-derived MSCs decreased their calcium deposition after 4 weeks in comparison with 2-week calcium deposition.

#### Osteoblastogenesis-associated gene expression analysis

**Fig. 9:**
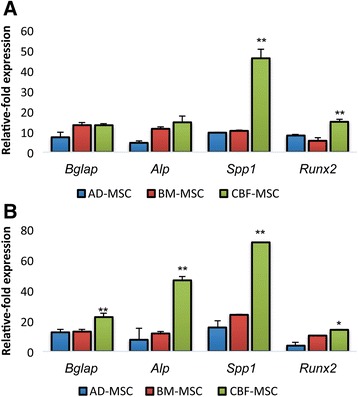
Quantitative polymerase chain reaction analysis for the expression of the osteogenic markers (*Bglap*, *Ssp1*, *Alp*, and *Runx2*) for AD-, BM-, and CBF-MSCs at 4 weeks of induction in normoxic (**a**) and hypoxic (**b**) conditions. Data are represented as fold-change compared with the expression levels found in the uninduced cells, respectively. Values are mean ± standard deviation (*n* = 3). Asterisks depict significant (*P* < 0.05) and highly significant (*P* < 0.01) differences between CBF-MSCs and AD- or BM-MSCs. *AD-MSC* adipose tissue mesenchymal stem cell, *BM-MSC* bone marrow mesenchymal stem cell, *CBF-MSC* cortical bone fragment

The expression of osteogenesis-associated genes confirmed the induction (Fig. 9). When induced, the three cell lines showed a marked upregulation of the tested genes. When CBF-MSCs were cultured in normoxic conditions, the expression levels increased to 13.21 (±1.01)-fold for *Bglap*, 14.66 (±2.99)-fold for *Alp*, 46.12 (±4.55)-fold for *Spp1*, and 14.92 (±1.22)-fold for *Runx2* compared with the uninduced counterparts. Significant differences in *Runx2* and *Spp1* gene expression were found in BM-MSCs and AD-MSCs exposed to normoxic conditions when expression levels were compared with those obtained from CBF-MSCs (Fig. 9a). The hypoxic conditions were generally more effective in inducing osteogenesis for the three cell lines (Fig. 9b). In particular, CBF-MSCs were found to be more prone to differentiate toward the osteogenic lineage in hypoxic conditions. An upregulation of the genes *Bglap*, *Alp*, and *Spp1* was observed, showing 22.50 (±4.55)-, 46.56 (±7.4)-, and 71.46 (±4.16)-fold increases compared with their uninduced counterparts. The hypoxic conditions seemed not to affect the expression of transcription factor *Runx2*.

## Discussion

Many tissues as sources of MSCs have been described, and for clinical applications, bone marrow is the most frequently used [50, 51]. As translation into practice becomes a reality, the ideal source for the intended application must be established.

Currently, the use of MSCs to promote bone regeneration in the clinical setting is limited to the use of bone marrow or iliac crest bone graft as sources of cells [51]. This has led researchers to identify alternative sources for acquisition of cells. Adipose has emerged as a reliable source because tissue can be easily obtained in larger quantities through lipoaspiration compared with bone marrow aspiration [30]. Based on this, several groups have compared the osteogenic potential of MSCs derived from the adipose tissue with that of MSCs obtained from bone marrow [31, 52–56]. Although results suggesting one source as favorable to another have been mixed [30–32], bone marrow overall was found to be superior as far as ALP activity, mineral deposition, and the expression of genes associated with osteogenesis are concerned [55]. Within the bone, besides the bone marrow [57], other tissues have already been proposed as sources of osteoprogenitor cells, including the cortical [17, 44, 58] and trabecular regions [16, 41–43]. The current literature, however, lacks of a direct comparison between human bone-derived presumptive MSCs in terms of osteogenic potential. Furthermore, a characterization under prolonged hypoxic conditions of CBF-MSCs or comparison of sources including compact bone under prolonged hypoxic conditions has not been reported. According to previously reported evidence for mouse [44] and rat [17] species, cells obtained represented a population which stained positively for the MSC-associated markers tested (CD44, CD105, and CD73) and negatively for the hematopoietic (CD45 and CD34) and the major histocompatibility-class II (HLA-DR) associated markers. In addition, once sorted, CBF-MSCs were analyzed for their expression of a recently described marker *CD271* useful to define an MSC precursor subpopulation, in comparison with the gold standard BM-MSCs, thus suggesting, also in humans, the cortical portion of the bone as a rich source of progenitor cells [59, 60]. Despite sharing a similar phenotype with MSCs derived from the bone marrow and the adipose tissue, however, when the osteogenic potential was assessed *in vitro* our findings suggested the cortical fraction of the bone as a superior source of osteoprogenitor cells to use in clinic for bone healing and bone repair. Compared with AD- and BM-MSCs, CBF-MSCs demonstrated higher activity of ALP following osteogenic induction after 14 days and a greater calcium deposition at both 2 and 4 weeks than their bone marrow counterparts. Results obtained from the evaluation of the ALP activity might suggest the compact bone as a source for MSCs may prime cells for an osteoblastic lineage [17], thus holding significant impact in the field of osteoregenerative tissue engineering. At a molecular level, our data support these observations showing a marked upregulation in *Alp*, *Runx2*, and *Spp1* expression. Specifically, *Alp* and *Runx2* are considered to play a critical role in the regulation of osteoblastogenesis, whereas *Bglap* and *Spp1* are associated with a more mature differentiation [61–63]. In our study, although the expression of *Bglap* was similar between BM-MSCs and CBF-MSCs, CBF-MSCs were found to express higher levels of *SSP1*, confirming the more mature osteoblastogenesis even compared with BM-MSCs [17]. Concomitantly with their marked osteogenic potential compared with the other sources, their reduced ability to form CFU-F corroborates the more committed nature of CBF-MSCs, according to previous evidence reported by us for rat [17]. Doubling time mean values further supported this statement, showing a generally slower proliferation rate for CBF-MSCs compared with the other cell lines in both normoxic and hypoxic conditions. As previously hypothesized for rat CBF-MSCs, the increased proliferation rate observed in human CBF-MSCs overtime might reflect the resting nature of osteoprogenitor cells lying on the bone surface, further confirming their superior osteogenic commitment. [17].

From the standpoint of translation, these findings have profound implications. One great downfall of transplanted stem cell therapeutics in tissue engineering is their inability to survive the harsh hypoxic environment of the recipient tissue [64–66]. Their ability to survive in an environment that is deprived of both oxygen and nutrient supply is necessary for successful tissue repair and angiogenesis [67]. Similarly, tissue engineering of cell-seeded constructs above a critical-size defect is often problematic because availability of oxygen and nutrient is limited to imbibition in the avascular recipient environment. Because of their increased and persistent biosynthetic activity in prolonged hypoxic conditions, the CBF-MSCs may be better suited for orthopedic tissue engineering of critical-size defects and other traumatic injuries compared with other sources of MSCs. One potential mechanism for the differences in osteogenesis seen here could be differential activation of hypoxia response pathways. Hypoxia-inducible factor (HIF) is the major regulator of cellular hypoxic responses and is known to be upregulated in MSCs [68]. Furthermore, it has been reported that HIF is essential for peripheral blood MSC mobilization seen in hypoxia [69]. A deeper understanding of the activation of hypoxia response pathways in the CBF-MSCs may elucidate the mechanisms leading to the enhanced biosynthetic activity and is the subject of our ongoing investigations in this cell population.

Our approach may not be suited for all orthopedic reconstruction as many times bone is not reamed or removed and thus a second procedure would be necessary. We believe, though, that in operative scenarios where, as part of the standard procedure, bone is exposed, resected, and discarded, fragments can be processed to isolate cells. Procedures such as spinal fusions, laminectomies, and traumatic orthopedic reconstruction would be ideal candidates for harvesting exposed bone tissue for cell isolation and potential re-implantation. Thus, the patient would be spared from additional procedures. These pieces previously thought of as waste house a population of cells with the potential for dramatic augmentation of bony regeneration without the need for an additional invasive procedure for the acquisition of tissue for processing and isolation of cells. More work needs to be done on the molecular characterization of these cells and how the effect of the tissue they originate from affects their commitment. As Shafiee et al. demonstrated with the preferred differentiation of AD-MSCs into adipocytes, this study represents a further example of how, regardless of phenotypic characterization prior to induction, the source the cells are derived from plays a critical role in their ultimate performance in translational applications [56]. Limitations to this and many other studies of its type are the conditions of cell culture used in this investigation. Generally speaking, the environment of intended use is three-dimensional (3D) with mechanical forces and cellular interactions [70, 71]. We acknowledge that to draw more relevant conclusions *in vitro*, culture conditions using bio-mimetic 3D scaffolds for growth should be sought.

In conclusion, our study demonstrates that quantitative differences between MSCs acquired from bone marrow, adipose, and the cortical portion of the bone exist with respect to their osteogenic potential. These differences are further augmented under conditions of prolonged low-oxygen tension. Our findings suggest the compact bone as a suitable candidate to replace bone marrow as the preferred cell source in selected situations of orthopedic tissue engineering and regenerative medicine. This confirms the presence of lineage predisposition in different stromal cell compartments that influences their ultimate differentiation and potential for use in clinical scenarios [72, 73]. Thus, the choice of cell source must be based on the intended surgical application and accessibility of patient tissue. In this setting, the identification of tissue-specific MSC epigenetic signature may help in the development of markers that are predictive of the *in vivo* biologic activity of MSCs and could potentially be used to screen MSCs prior to their use.

## Conclusions

Although MSCs from various sources have been identified, the optimal source for orthopedic regeneration has yet to be identified. The cortical fraction of the bone has been shown to house a homogeneous population of cells with distinct immunologic and phenotypic characteristics. Compared with BM-MSCs and AD-MSCs, CBF-MSCs retain superior osteogenic potential in both normoxia and hypoxia. Altogether these properties may contribute to enhance regenerative potential in the harsh microenvironments of tissue engineered constructs and healing critical-size defects. More research needs to be done *in vivo* to characterize this cell source as the optimal source for orthopedic regeneration.

## Abbreviations

3D: Three-dimensional
α-MEM: Alpha-modified Eagle’s medium
AD-MSC: Adipose tissue mesenchymal stem cell
ALP: Alkaline phosphatase
Bglap: Osteocalcin
BM-MSC: Bone marrow mesenchymal stem cell
CBF-MSC: Cortical bone fragment- mesenchymal stem cells
CFU-F: Colony-forming unit-fibroblastic-like
DMEM: Dulbecco’s modified Eagle’s medium
FACS: Fluorescence-activated cell sorting
FBS: Fetal bovine serum
H&E: Hemotoxylin and eosin
HIF: Hypoxia-inducible factor
MHC: Major histocompatibility complex
MSC: Mesenchymal stem cell
P: Passage
PBS: Phosphate-buffered saline
Runx2: Runt-related transcription factor 2
Spp1: Osteopontin
